# Could the minimum intervention oral care framework help improve the quality of oral health delivery and access to NHS primary dental care?

**DOI:** 10.1038/s41415-024-7627-x

**Published:** 2024-08-14

**Authors:** Lamis Abuhaloob, Austen El-Osta, Tim Newton, Salman Rawaf, Avijit Banerjee

**Affiliations:** 41415155391001 WHO Collaborating Centre for Public Health Education and Training, Primary Care and Public Health, School of Public Health, Imperial College London, 90 Wood Lane, W12 0BZ, UK; 41415155391002 Self-Care Academic Research Unit (SCARU), Imperial College London, Department of Primary Care and Public Health, Imperial College School of Public Health, 90 Wood Lane, W12 0BZ, UK; 41415155391003 Centre of Oral Clinical Translational Sciences/Conservative & MI Dentistry, Faculty of Dentistry, Oral and Craniofacial Sciences, King´s College London, Great Maze Pond, London, SE1 9RT, UK

## Abstract

Access to NHS primary dental care services is a perennial issue in the UK. Two aspects must be considered when measuring access to dental care: ‘entry access', which relates to service availability resulting in realised initial or continued access; and ‘effective access', the delivery of effective, equitable and efficient care, which manifests as equitable and optimal outcomes of care. It is proposed that the minimum intervention oral care (MIOC) delivery framework provides a person-focused, prevention-based, susceptibility/needs-related, team-delivered approach to ensuring effective access to primary oral and dental care. A theory of change model could identify the key barriers to overcome the implementation of the MIOC approach, involving all key stakeholders in primary oral and dental care delivery.

## Key points

- The issue of NHS dental care access continues to worsen.
- Access to NHS oral and dental care services is influenced by the interaction between the healthcare system, patients' circumstances and population characteristics.
- Theoretically, the integration of a prevention-based, susceptibility/needs-related, person-focused minimum intervention oral care (MIOC) framework into NHS primary dental care could improve access to care for those that most need it while maintaining optimal care outcomes.
- A theory of change model could identify the key barriers to implementing the MIOC approach in the NHS primary oral and dental care delivery.

## Background

###  Accessing NHS primary oral and dental care services

The English NHS provides essential treatments for teeth, periodontal tissues and the mouth, including free oral and dental treatment for people under 19, pregnant people and 12 months after birth, patients whose treatment is carried out by a hospital dentist after being treated in an NHS hospital, those receiving low-income benefits and dependants under 20 years old.^1^

To develop effective and equitable delivery of dental care services, since 2006, the dentists having NHS contracts have been paid to provide an agreed level of dental activity each year, measured in units of dental activity (UDAs) from April until the following March, paid in 12-monthly instalments. The NHS recovers the unused funds if the dentist does not fulfil 96% or more of the agreed UDAs. However, if these units run out, patients have to go elsewhere or wait until the NHS practice receives its new quota. Since 2011, there have been many pilots within the dental contract reform programme to improve the contracts and commissioning with dentists to improve access to oral and dental care. This programme ended on 31 March 2022 and all prototype contracts returned to follow the contractual terms and conditions of general dental service or personal dental services.^2^

Accessibility to healthcare is defined as the opportunity to reach and/or obtain the healthcare services that fulfil the seekers' needs.^3^^,^^4^^,^^5^^,^^6^ Evaluating access to healthcare is a complex process^3^^,^^7^^,^^8^^,^^9^ but most researchers have agreed that the ultimate access to healthcare is a collective outcome that stems from the interface between five accessibility dimensions of healthcare service provision: i) approachability; ii) acceptability; iii) availability and accommodation; iv) affordability; and v) appropriateness.^2^ These dimensions are influenced by the individuals' abilities to obtain needed services (ie to perceive; to seek; to reach; to pay; to engage), as well as determinants related to the characteristics of individuals, the community and health system and/or service providers. These determinants are the principal barriers and/or facilitators for accessing healthcare.^3^^,^^4^^,^^5^^,^^10^

Harris (2013)^8^ proposed that measurement of patient access could be realised by assessing two metrics incorporating four measurable constructs:

- Entry access - defined as ‘whether individuals and groups can receive initial care', determined and influenced by measuring two constructs: i) service availability and ii) realised initial and continued access
- Effective access - defined as ‘the proportion of the population in need of an intervention that receives an effective intervention', determined by: iii) equity and iv outcome of care constructs.

Several UK-based studies identified different factors that influence access to NHS oral and dental care services (Table 1). Some factors are related to the healthcare system (eg structural barriers)^11^^,^^12^^,^^13^^,^^14^ whereas others are associated with patients' circumstances and population characteristics (eg personal barriers).^13^^,^^15^^,^^16^^,^^17^^,^^18^

**Table 1:** Shortcomings in quantity, equity and quality of NHS oral and dental care access

Shortcomings in quantity and equity (barriers to entry access)	Shortcomings in equity and quality (barriers to effective access)
Shortage in dentist and oral healthcare team members' availability,^37^^,^^38^ contemporary training in prevention^13^ and awareness of the NHS initiatives to improve NHS oral and dental care access^32^ Rising costs of oral and dental care^2^^,^^37^ Unmet oral and dental care needs of vulnerable children and older adults mainly living in socio-economic deprivation^20^^,^^21^^,^^30^ Lower use of NHS oral and dental care services by those living in socio-economic deprivation and low- and middle-income groups^15^^,^^17^	Dentists' failure to prioritise quality over quantity in oral and dental care provision because of the fee-for-service remuneration systems in place^28^ Impact of health system and professional regulations on NHS oral healthcare team members' health and wellbeing and dental care quality provision^31^ Failure to deliver consistent, person-focused, prevention-based care, with longitudinal review of susceptibility/need as an indicator of quality of oral and dental care provision^26^ Variability in policymakers' views about NHS oral and dental healthcare services in the four devolved regions of the UK^27^ Low patient and carer perceptions and awareness of NHS oral and dental care mainly for high-risk/susceptibility and high-needs patient groups^13^ Inequities in NHS oral and dental care provision according to social grade, ethnicity, sex and age.^35^^,^^36^ Public Health England reported inequities in the availability and utilisation of oral and dental services across various age groups, sex, geographical location and different socio-economic groups.^18^

Entry access to NHS primary dental care services for patients is a perennial issue in the UK.^6^^,^^8^^,^^19^^,^^20^^,^^21^ Even prior to the COVID-19 pandemic, O´Connor *et al*.^22^ declared shortfalls in entry access to NHS dental care for children and adults, as 58.4% of children and 49.6% of adults were seen by an NHS dentist in 2019.^23^ Subsequently, the British Dental Association estimated that over 38 million dental appointments were missed over the course of the pandemic alone.^2^ Despite efforts by the NHS to promote oral and dental care quality and access, the problem of NHS dental care access continues to worsen and has exacerbated since the advent of the COVID-19 pandemic.^2^ In the UK, the lack of NHS dental care access or delayed dental treatment has led to increased patient pain, destruction of tooth tissue, and in severe cases, infections and worsening dental and periodontal status, with subsequent potentially avoidable tooth loss.^24^

Despite some improvement in entry access to NHS oral and dental care shown by the GP Patient Survey in 2022/2023, the UK House of Commons stressed that the success rate in securing NHS dental care appointments is still below a 92% success rate compared to 2019.^25^^5^

Shortcomings in the equity and quality of the NHS dental care services (effective access) have been attributed to multiple factors, as summarised in Table 1, and vary between impacts of the current NHS system, professional regulations and policymakers' views on the provision of person-focused, prevention-based and sustainable care,^26^^,^^27^ whilst also impacting on dentists' productivity and wellbeing.^28^ Further factors are patients' low perception and awareness of NHS oral and dental care provision^13^ and inequities in NHS oral and dental care provision for different age, ethnic, sex and social groups.^18^^,^^29^^,^^30^ More recently, a semi-structured interview of 20 dentists in England found that they faced multiple factors that negatively influenced their physical, psychological and emotional health and consequently, their dental care quality provision.^22^ These factors were related to professional regulations, health systems, job specifications, relationships and personal life.^31^

Few studies have investigated the enablers and facilitators of better effective oral and dental care access in the NHS. One study considered using NHS Direct to promote equitable 24-hour and out-of-hours access to NHS dental care.^32^ Another study looked at initiating direct access by the General Dental Council in 2013 to allow dental hygienists and dental therapists to treat patients independently without treatment prescriptions from a dentist first.^28^^,^^29^ A third study considered how the NHS welfare system effectively reduced the negative impact of social differences in oral health improvement and reduced inequalities in dental care access in the UK.^33^^,^^34^ The Fuller Stocktake report^34^ also considered the potential positive role of the NHS 111 (out-of-hours on-call) and direct access integration into primary and urgent care routine access. Yet, in 2017, concerns were raised by General Dental Council-registered dental hygienists and dental therapists offering direct access centred around the lack of dental nurse support and the limited availability of periodontal treatment under NHS regulations.^35^ Consequently, NHS England produced guidance for the use of skill mix within NHS general dental practice^36^ and clarified dental therapists' and dental hygienists' roles within the oral healthcare team in providing patient care within NHS primary dental services (eg diagnostic and treatment work) through direct access.

## Minimum intervention oral care as a solution to effective access

Undoubtedly, developing a prevention-based, susceptibility-related, cost-effective, long-term model of oral and dental healthcare delivery to sustainably solve the dental care access conundrum is urgent and highly needed.^34^^,^^39^ This is aligned with the principles of the Long-Term Plan set by the UK government and NHS England for delivering better oral health and ensuring better access to oral and dental care for all.

The minimum intervention oral care (MIOC) delivery approach may offer a tenable solution to the problem of effective access to NHS oral healthcare. The prevention-based, susceptibility/needs-related, person-focused MIOC framework delivered to patients by oral healthcare teams, with essential two-way interaction between the team and patient/caregiver (shared decision-making), combines the four key clinical domains of: 1) detection and diagnosis (the identification of patients' problems to enable appropriate susceptibility assessments, detection, diagnosis, prognosis and phased personalised care planning); 2) prevention of lesions and control of oral and dental disease (primary and secondary prevention of lesions/control of disease using non-operative or micro-invasive interventions and patient preventive behaviour modelling techniques); 3) minimally invasive operative interventions (tertiary prevention) to treat developing carious and periodontal lesions and to manage and prevent their negative impact (dental cavities, fractured/broken down teeth, pulp and periodontal pathology) on oral and general health; and 4) review/recall/active surveillance to help the patient maintain life-long optimal oral and dental health with longitudinal susceptibility assessment.^40^^,^^41^^,^^42^^,^^43^^,^^44^^,^^45^^,^^46^^,^^47^^,^^48^^,^^49^^,^^50^^,^^51^^,^^52^

Given the challenges, an important question is whether applying the MIOC framework in the NHS primary dental care service could improve access to care for those who most need it, whilst maintaining optimal care outcomes. This could potentially be achieved by exploring three benefits of MIOC. Firstly, this approach could help change oral healthcare team and patients' behaviours and support more sustainable, long-term oral and dental patient self-care to prevent lesions and control disease (namely dental caries and periodontal disease, as well as tooth wear), consequentially reducing demand for current NHS Band 2 and 3 treatments over time. Secondly, MIOC will help in identifying those high-susceptibility/high-needs patient groups, encouraging targeted preventive oral and dental care behaviours and applying relevant micro-invasive and minimally invasive interventions where required. Thirdly, this approach will help in re-assessing and performing team-delivered active surveillance (in-person or remotely) for risk-reduced, sustainable, oral health maintenance, mainly for those most in need (children, older adults and those living in socio-economic deprivation), ensuring optimal clinical care outcomes and improved patient satisfaction.

Indeed, best clinical practice supports MIOC's efficiency in managing oral diseases and enabling oral healthcare teams to deliver long-term, high-quality oral healthcare.^46^^,^^53^ The challenge lies in the realisation that the uptake of MIOC is not universal in primary care and there are still concerns about how the MIOC principles can be effectively implemented and remunerated within current primary care practice systems/business models.^43^ Banerjee (2013)^41^ proposed that the MIOC approach is underpinned by ‘the interactive (shared decision-making) team-care approach to patient management'. This model prioritises non-operative disease control and prevention services over often unnecessary, expensive and inappropriate operative interventions. Consequently, if applied at scale, the demand for dental care access will be reduced and better targeted, with patient care outcomes and satisfaction seeing an upward trend. Thus, the relevant stakeholders, including dental service providers, government healthcare regulators and dental industry partners, should all collaborate with clinical teams, teachers and clinical research academics to integrate MIOC into the NHS mainstream and empower dentists and oral healthcare team members to maintain effective delivery of person-focused care services.^41^

**Fig. 1:**
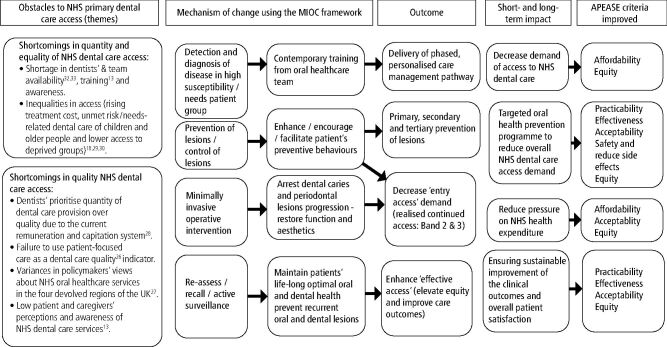
MIOC theory of change based on identified barriers from the literature

In principle, MIOC can prevent the incidence of new oral and dental diseases (mainly among people at higher risk/susceptibility of suffering dental diseases), make more efficient use of team-delivered time for targeted prevention-based and phased care provision, and in so doing, can help reduce the pressure in demand for access to oral and dental care.^53^ MIOC also ensures high-quality care outcomes, with clinical outcome measures based on disease and susceptibility reduction/prevention, whereas focusing its application on vulnerable patient groups could also help reduce inequity in care provision.^54^ Further clinical studies in primary care settings are required to verify these logical and pragmatic assumptions. Figure 1 depicts the theoretical framework for the feasibility of MIOC implementation in improving access to NHS oral and dental care.

## Implementing MIOC - a theory of change

The interactional relationship between the available NHS oral and dental care system, policies, decision-makers' views, oral healthcare team members' wellbeing and productivity, and patients' demands for accessing high-quality dental care, all together determine the current outcome of access and quality of dental care received by patients.

The NHS plan prioritises patient behaviour change based on the COM-B model (capability, opportunity and motivation capable of changing behaviour) to promote sustainable, long-term self-care skills, preventing the onset of new disease and manage/control existing diseases.^55^ The COM-B model suggests that providing all patients with the capability, opportunities and suitable goal-setting motivation to adopt more healthy behaviours would mitigate oral as well as general disease susceptibilities.^34^^,^^39^^,^^55^

**Fig. 2:**
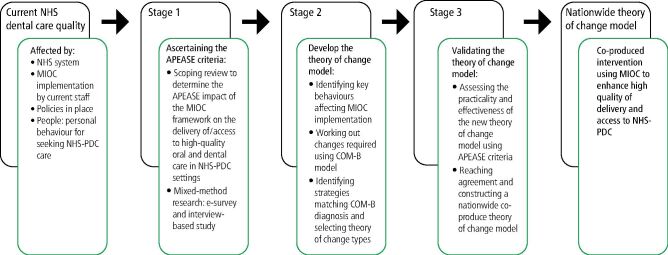
Project methods flowchart and theory of change model for enhancing the quality of oral health delivery in primary dental care (PDC) using MIOC framework

The application of the behaviour change wheel model can guide the implementation of interventions to adapt the existing MIOC framework for the NHS-PDC nationwide. Figure 1 and Figure 2 depict the process to implement the theory of change and test MIOC implementation feasibility, accessibility and practicability in achieving the desirable behaviours and enhancing the delivery of/access to high-quality oral and dental care.

The assessment of the appropriateness of existing MIOC intervention in NHS primary dental care in terms of the APEASE criteria - acceptability, practicability, effectiveness, affordability, side-effects and equity - could be carried out. This would help to identify and map the interaction of existing barriers and behaviours that influence MIOC performance and/or fail to meet one or more of the APEASE criteria.^56^

The ultimate goal of this discussion paper is to introduce the concept of enhancing the quality of oral and dental healthcare services delivery in primary care using the MIOC delivery framework, which has been shown to be effective in improving the quality of care in secondary dental care settings and for at-risk patients.^46^ Figure 2 is based on the theoretical assumption that the nationwide implementation of MIOC in NHS primary dental care will enhance the quality of dental care delivery and access using the improvement APEASE criteria as a parameter for assessment.

## Conclusion

To summarise, ensuring effective access to NHS oral and dental primary care is a multi-factorial, complex issue. NHS dental access is influenced by national (political and economic), institutional and community cross-interactions. Thus, a prevention-based MIOC delivery framework could enable the NHS to provide a sustainable, cost-effective, long-term model of dental care. Although MIOC has been piloted in NHS secondary dental care, this paper concludes with a call to action by proposing the theoretical assumption of MIOC's potential effect in increasing ‘effective access' and potentially reducing ‘entry access' pressure in the NHS primary dental care service in the UK. Further to this call for action, we also make the case for using the behaviour change wheel theory to develop a nationwide intervention to improve the quality of care delivery and access in the NHS primary dental care settings using the MIOC framework.
